# Advances in molecular imaging of immune checkpoint targets in malignancies: current and future prospect

**DOI:** 10.1007/s00330-018-5814-3

**Published:** 2018-11-30

**Authors:** Yang Du, Yinhua Jin, Wei Sun, Junjie Fang, Jianjun Zheng, Jie Tian

**Affiliations:** 1 CAS Key Laboratory of Molecular Imaging, The State Key Laboratory of Management and Control for Complex Systems, Institute of Automation, Beijing, 100190 China; 2 Beijing Key Laboratory of Molecular Imaging, Beijing, 100190 China; 3 University of Chinese Academy of Sciences, Beijing, 100080 China; 4 Department of Radiology, Ningbo No.2 Hospital, Xibei Street 41#, Haishu Dist., Ningbo, 315010 Zhejiang China; 5 Beijing Advanced Innovation Center for Big Data-Based Precision Medicine, Beihang University, Beijing, China

**Keywords:** Cancer, Immunotherapy, Molecular imaging, Immune checkpoint target

## Abstract

**Objectives:**

This review describes the current status and progress of immune checkpoint targets for imaging of malignancies. Immune checkpoint blockade holds great potential for cancer treatment, and clinical implementation into routine is very rapidly progressing. Therefore, it is an urgent need to become familiar with the vocabulary of immunotherapy and with the evaluation of immune checkpoint and related treatments through noninvasive molecular imaging. Currently, immune target-associated imaging mainly includes PET, SPECT, optical imaging, and MRI. Each imaging method has its own inherent strengths and weaknesses in reflecting tumor morphology and physiology. PD-1, PD-L1, CTLA-4, and LAG-3 are the most commonly considered targets. In this review, the current status and progress of molecular imaging of immune checkpoint targets are discussed.

**Conclusion:**

Molecular imaging is likely to become a major tool for monitoring immunotherapy. It can help in selecting patients who are suitable for immunotherapy, and also monitor the tumor response.

**Key Points:**

*• Immune checkpoint blockade holds great promise for the treatment of different malignant tumors.*

• *Molecular imaging can identify the expression of immune checkpoint targets in the tumor microenvironment at the molecular and cellular levels, and therefore helps selecting potential responders, suitable for specific immunotherapy.*

• *Molecular imaging can also monitor immunotherapeutic effects, and therefore participates in the evaluation of tumor response to treatment.*

## Introduction

Immune checkpoints (IC) refer to inhibitory pathways in immunoreactions that are momentous for self tolerance. These pathways can suppress T cell effector function leading tumors to evade immune surveillance [1, 2]. IC inhibitors targeting programmed cell death receptor 1 (PD-1) and its ligand (PD-L1), cytotoxic T lymphocyte-associated protein 4 (CTLA-4), and lymphocyte activation gene-3 (LAG-3) are over-expressed in several cancers, such as lung cancer [3], melanoma [4], and triple-negative breast cancer (TNBC) [5]. As summarized in Table 1, some IC target inhibitors have been tested in clinical trials. It is reported that IC as PD-1/PD-L1 expression is associated with the poor prognosis of tumors and also with the efficacy of immunotherapy [6].

**Table 1:**
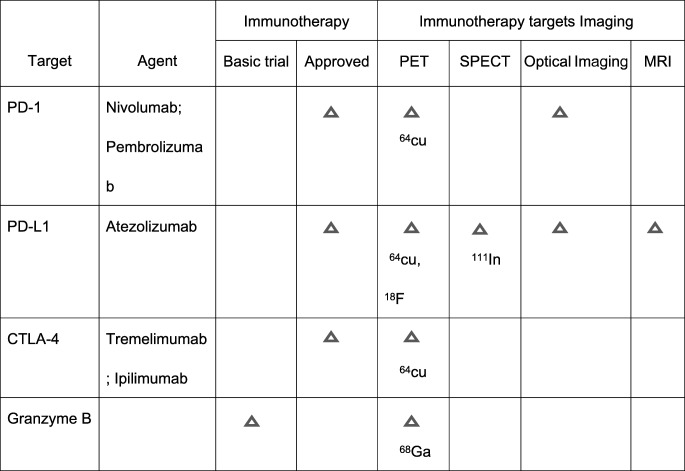
The application of immune checkpoints in clinical trials and the imaging of immune checkpoint targets in malignancies

**Fig. 1:**
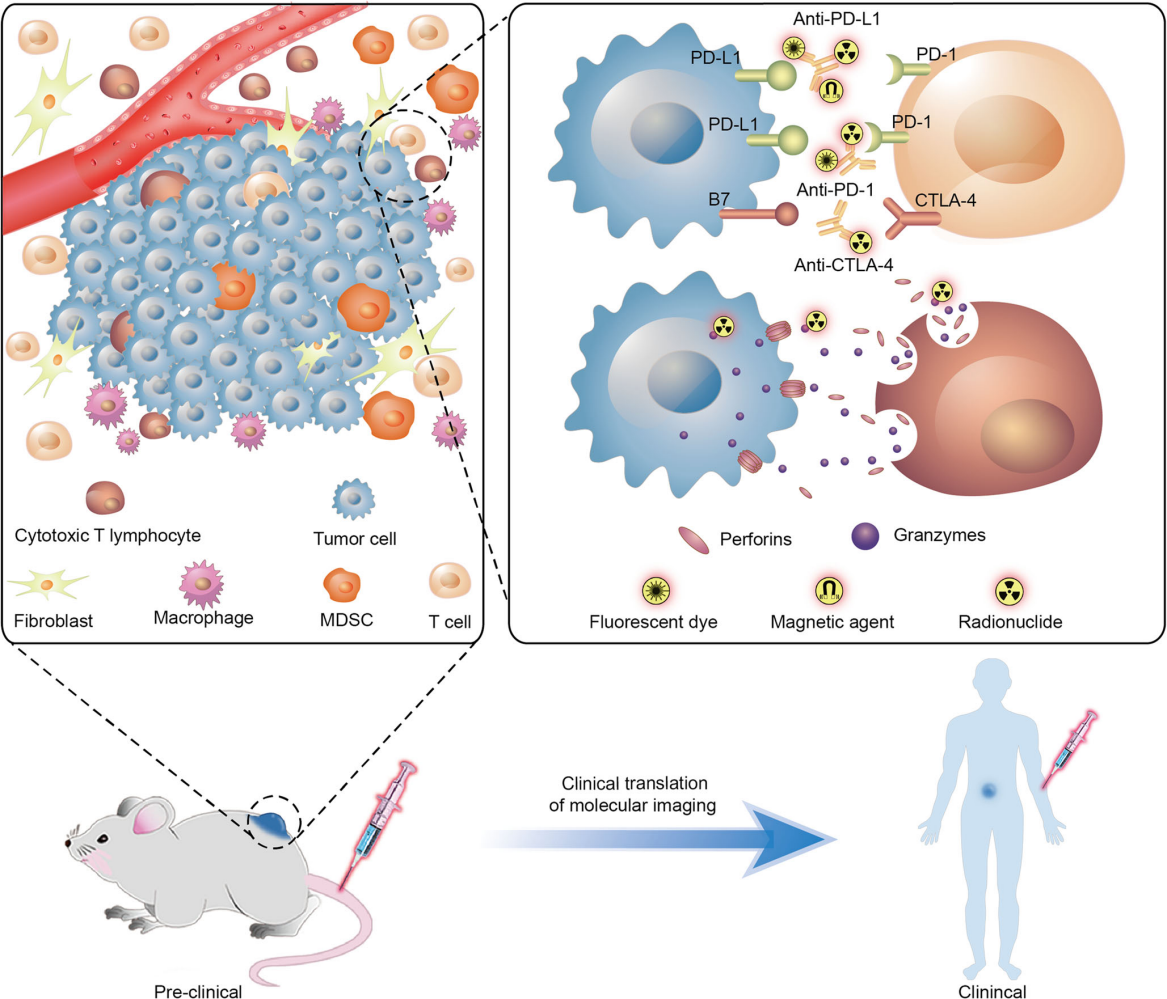
Targeted molecular imaging of immune checkpoints from preclinical to clinical studies. In tumor micro-envirenment, radionuclide, fluorescent dye, or magnetic agent labeled monoclonal antibodies as anti-PD-L1, anti-PD-1, anti-CTLA4 et al were performed using SPECT, PET/CT, MRI, or optical imaging. Cytotoxic T lymphocytes were activated by immune checkpoint blocking treatment causing a higher releasing of granzyme B; radionuclide-labeled granzyme B was utilized as a target for PET imaging

Molecular imaging is a real-time approach of tumor biomarkers that can accurately monitor the dynamic changes of the target expression and differentiate tumor from normal tissue [7]. The specific radionuclides or optical probes have been developed for the visualization of the immunotherapeutic targets at the molecular and cellular levels. Moreover, molecular imaging can be used for repeated assessment of the same individual before and after the treatment. Immune checkpoint target-associated imaging in the current basic research mainly includes MRI, PET, SPECT, and optical imaging [8]. As every modality has its own strengths and limitations, multimodality imaging is considered to be potentially more powerful. In this review, the current status and future directions of molecular imaging of IC targets on malignancies are discussed (Fig. 1).

## PD-1

**Fig. 2:**
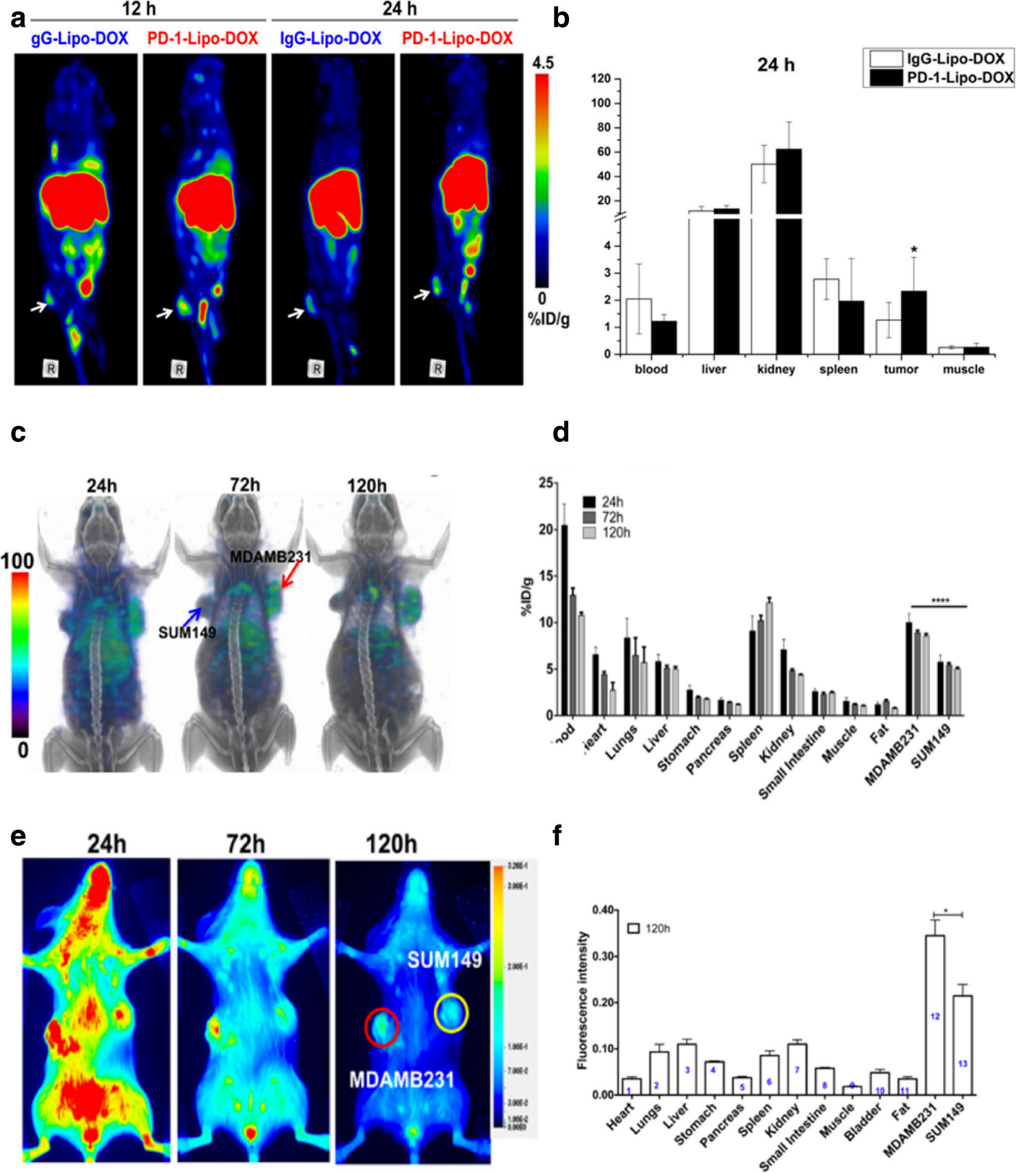
Multimodality molecular imaging of PD-1/PD-L1 expressing cancer. **a**, **b** PET images of 4T1 mammary tumor-bearing mice at 12 and 24 h postinjection of PD-1-Liposome-DOX-DOTA-^64^Cu, and biodistribution of PD-1-Liposome-DOX-DOTA-^64^Cu in 4 T1 tumors 24 h postinjection. **c**, **d** SPECT images were acquired at 24, 72, and 120 h after the injection with 14.8 MBq (400 uCi) of ^111^In-PD-L1-mAb. The SPECT images showed the higher intensity biodistribution of ^111^In-PD-L1-mAb in the MDA-MB-231 tumor compared to SUM149 tumor in the same tumor-bearing mice, and ex vivo biodistribution analysis of [^111^In] radioactive tracer intensity in the different tissues at 24 h, 72 h, and 120 h postinjection. **e**, **f** Optical images showed specific fluorescence biodistribution of NIR-PD-L1-mAb in the MDA-MB-231 tumor compared to SUM149 tumor in the same tumor-bearing mice, and the ex vivo biodistribution analysis of fluorescence intensity in the different tissues at 120 h postinjection (**a** and **b** from Du, with permission of [14]. **c**–**f** [15] by Chatterjee S is licensed under CC BY 3.0)

PD-1 is an immunosuppressive receptor expressed on immune cells, including activated T cells, regulatory T cells, B cells, monocytes, and dendritic cells (DCs), probably due to stimulation by chronic antigens [9]. There are two PD-1 ligands: PD-L1 and PD-L2. PD-L1 is more widely expressed in cancers than PD-L2. The PD-L1/PD-1 interaction results in the inhibition of T cell activation [10]. PD-1/PD-L1 signaling pathway not only inhibits the activation and function of CD8^+^ T cells but also enhances the tumor immunosuppressive environment by regulatory T cells (TREGS) [11]. Nivolumab and pembrolizumab are anti-PD-1 mAbs that have been approved by the US Food and Drug Administration (FDA) for the clinical treatment of metastatic melanoma and non-small cell lung cancers [12]. It has been elucidated that PD-1^+^/FOXP3^+^ TREGS were detected in the tumor microenvironment, and that PD-1/PD-L1 expression was correlated with poor prognosis of tumors [13]. Hence, PD-1 represents a potential immune target for molecular imaging in cancers. Studies have reported that PD-1 expression can be detected with ^64^Cu-labeled anti-PD-1 mAb on tumor-infiltrating lymphocytes (TILs) of B16-F10 melanoma tumors using PET scans. PET imaging has been demonstrated to possess a prognostic value for immunotherapy of PD-1 checkpoint blockade [11]. This was the foundation for PD-1-targeted imaging. Moreover, Du et al synthesized a dual PET and optical dual imaging agents labeled anti-PD-1 mAb, anti-PD-1 mAb-labeled liposomes conjugated with IRDye800CW, and ^64^Cu-DOTA to image mouse breast 4T1 tumor with near-infrared fluorescence imaging and PET [14]. A relatively higher PET signal was found in the anti-PD-1 mAb-targeted group compared to the IgG control group, which was consistent with the near-infrared fluorescence (NIRF) imaging (Fig. 2a, b). The data suggested that anti-PD-1 mAb-targeted nanoparticle can effectively target the PD-1 expressing TILs in breast tumor [14].

## PD-L1

PD-L1, also called CD274, is an immunoinhibitory molecule that suppresses the activation of T cells when binding to the PD-1. It is not only expressed on the surface of tumor cells, but also on antigen-presenting cells in various solid malignancies [16, 17]. There is a close correlation between PD-L1 expression and poor prognosis of tumors, especially the digestive system malignancy, urogenital neoplasms, and TNBC [18]. PD-L1-targeted imaging is most frequently studied in malignancies to quantify PD-L1 expression.

### SPECT/PET-CT imaging of PD-L1 expression

Heskamp S et al investigated the biodistribution of PD-L1.3.1 mAbs through radiolabeling with indium-111 (^111^In) for SPECT/CT imaging. ^111^In-PD-L1.3.1 specifically bound to the high PD-L1-expressing TNBCs (MDA-MB-231 cells) with a heterogeneous distribution [19]. The highest accumulation was observed in the periphery of MDA-MB-231 tumors, whereas the lowest accumulation was found in the tumor center with necrosis. Moreover, ^111^In-PD-L1-mAb was monitored for specificity using five breast cancer cell lines, and different PD-L1 expression levels were observed (Fig. 2c, d). It has been proven that PD-L1-targeted SPECT/CT imaging is a viable option that can predict the anti-PD-L1 IC therapeutic response in TNBC [15].

PET imaging also provides a noninvasive approach to assess the pharmacokinetics of radiolabeled antibody drugs, which exhibit high affinity for specific antigens. PET imaging commonly used radionuclides that include ^64^Cu (half-life of 12.7 h), ^68^Ga (half-life of 68.1 min), and ^89^Zr (half-life of 3.7 days). Atezolizumab is an anti-PD-L1 antibody used for treating several malignant tumors like non-small cell lung cancer [20], melanoma [21], and TNBC [22]. Lesniak WG et al reported the biodistribution and specific expression of PD-L1 in breast cancer cells using ^64^Cu-labeled atezolizumab with PET/CT imaging [23]. PET imaging also detected a significantly increased uptake of ^64^Cu-labeled atezolizumab in MDA-MB-231 tumors compared to SUM149 tumors (low PD-L1-expressing TNBC).

A recent study also showed in vivo dynamic PET imaging of ^18^F-radiolabeled affibody ligands (NOTA-ZPD-L1_1) in PD-L1-expressing melanoma tumors. The results showed a rapid uptake of the tracer in the PD-L1-positive tumors [24]. Donnelly et al showed PET imaging of mice bearing bilateral PD-L1-positive human lung malignant tumor and PD-L1-negative colon malignant tumor with ^18^F-BMS-986192 (^18^F-fluorine labeled anti-PD-L1 adnectin). In vivo PET imaging showed a 3.5-fold higher uptake in PD-L1-positive lung tumor than PD-L1-negative tumor 2 h after injection [25]. Maute et al developed high affinity consensus (HAC) PD-1, a 14-kD protein with high affinity to human PD-L1, and radiolabeled with ^64^Cu as a PET imaging tracer to measure the expression of PD-L1 on CT-26 colorectal tumor-bearing mice [26]. Mayer et al developed six HAC-PD-1 radiotracer variants to detect human PD-L1 expression, and the data showed that ^64^Cu-NOTA-HACA-PD1 was the best tracer for monitoring the human PD-L1 expression in vivo. The uptake of ^64^Cu-NOTA-HACA-PD1 was significantly increased in the PD-L1-positive CT26 tumor and decreased in the nonspecific tissues, but it stays in vessels for a longer time. In order to decrease the blood clearance time, they found that the short half-life ^68^Ga variants can significantly reduce liver signal when compared with ^64^Cu variants because ^68^Ga is known to accumulate in the bone. This study may promote translation of IC imaging into clinical routine [27].

Hettich et al also reported noninvasive PET imaging of PD-1 and PD-L1 tracers in melanoma. Evaluation of ^64^Cu-NOTA-PD-L1 mAb uptake was performed in mice bearing PD-L1-positive B16F10 melanoma cells and PD-L1-deficient B16F10 cells on the opposing flank. Uptake of ^64^Cu-NOTA-PD-L1 tracer was detected in the region of PD-L1-positive melanoma, as well as the spleen, the lymph nodes, and the brown adipose tissues, but not in the PD-L1-deficient melanoma [28].

### Optical imaging of PD-L1 expression

Optical imaging is widely used to image the abnormalities at the molecular, cellular, and tissue levels in both preclinical and clinical settings. Fluorescent proteins and dyes play important roles in fluorescent molecular imaging studies. The Licor800 dye-conjugated PD-L1-mAb (NIR-PD-L1-mAb)-based imaging probes were utilized to monitor PD-L1 expression in different breast cancer cells. Samit et al found higher fluorescence signal intensities with NIR-PD-L1-mAb in MDA-MB-231 tumors (27% PD-L1-positive tumor cells) compared to SUM149 tumors (0.1% PD-L1-positive cells) (Fig. 2e, f). It demonstrated that the PD-L1-positive expression in TNBC can be specifically detected using a NIR-PD-L1 mAb probe [15].

### Optical and MRI dual-modality imaging of PD-L1 expression in tumor

Fluorescence and MRI imaging have the capacity to complement each other. To compensate the insufficiency of each imaging modality, Du et al performed dual-modality MRI and optical fluorescence imaging of PD-L1 expression in breast tumors. Du et al developed a novel theranostic nanoprobe, a PD-L1 mAb-targeted nanoparticle labeled with MRI contrast agent of Gd-DOTA and NIRF fluorescence dye (PD-L1-PCI-Gd). The fluorescence imaging revealed constantly higher fluorescent intensity in PD-L1-targeted nanoparticles in 4T1 tumors compared to the non-targeted control group. The tumor also showed approximately 2-fold higher PD-L1-targeted fluorescence intensity than the background. Similarly, MRI imaging revealed significantly higher signal intensity clearly and persistently in the 4T1 tumors compared to the control group [29].

## CTLA-4

CTLA-4 has been identified as the first IC receptor, also called cluster of differentiation 152 (CD152). CTLA-4 is an immune-inhibitory checkpoint that suppresses T cell-mediated immune responses, leading to the development of tumors. CTLA-4 has naturally become a clinically relevant target for imaging that relies on the activation and biodistribution of T cells in vivo. CD28 (cluster of differentiation 28) is one of the proteins expressed on the surface of T cell that promote T cell activation; CTLA-4 and CD28 possess identical ligands: CD80 and CD86 (B7.1 and B7.2). CTLA-4 has a higher affinity for CD80 and CD86 than CD28 that prevents and inhibits the interactions between B7 molecules and CD28 and dampens T cell activation, leading to the inhibition of intracellular signaling [30]. Ipilimumab, an anti-CTLA-4, was approved by the FDA for the treatment of metastatic and high-risk resected melanoma in 2011, and tremelimumab is currently under investigation as another potential anti-CTLA-4.

**Fig. 3:**
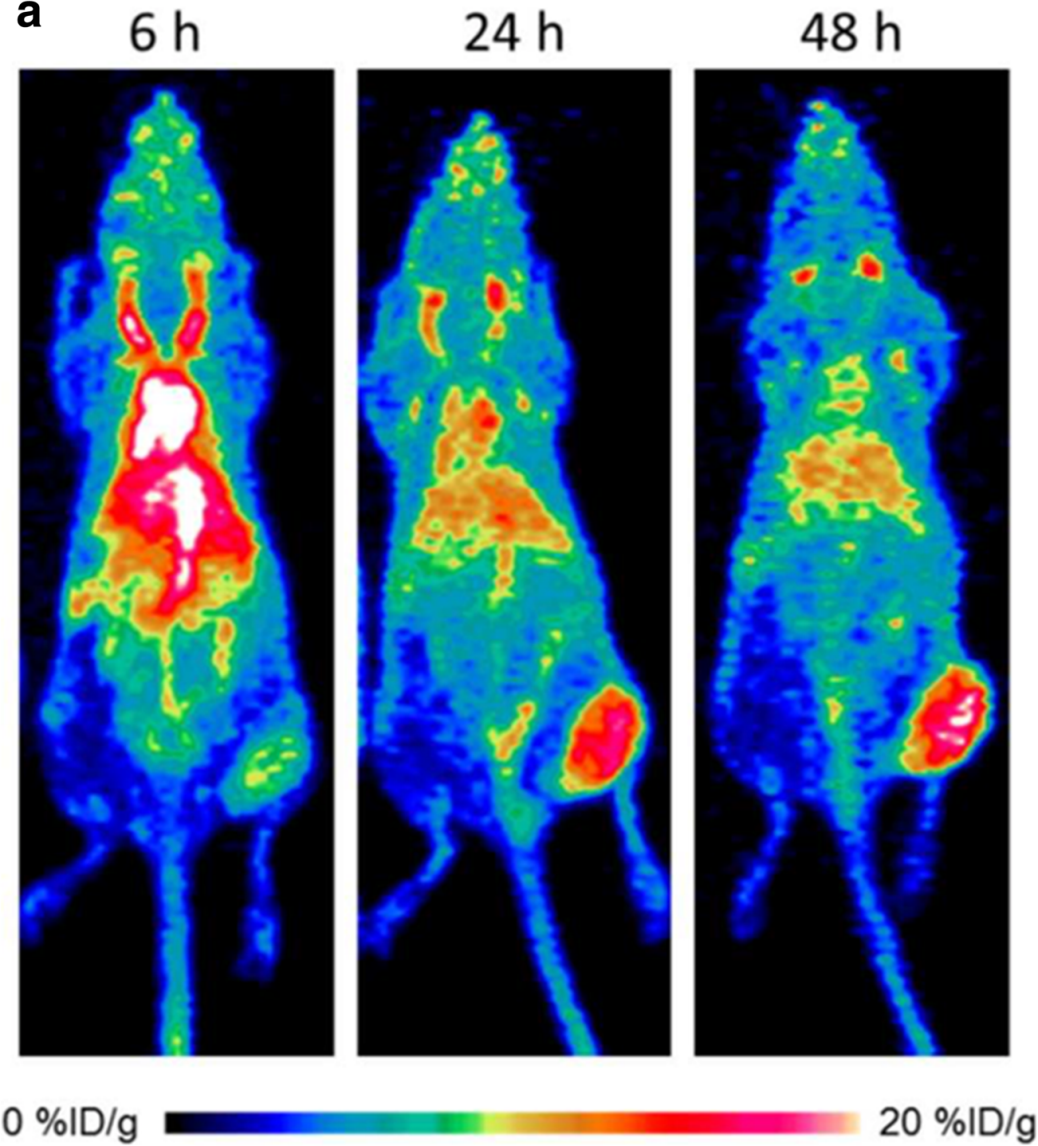
Representative maximum intensity projection images of longitudinal PET imaging of CTLA-4-expressing A549 lung cancer. PET imaging was acquired at 6, 24, and 48 h after injection with ^64^Cu-DOTA-ipilimumab. PET imaging showed the higher intensity in lung cancer (from Ehlerding EB, with permission of [32])

Initially, Higashikawa et al developed a radionuclide-labeled murine CTLA-4 antibody for imaging CTLA-4-expressing TILs in CT26 tumors. PET imaging was performed to examine the biodistribution and pharmacokinetic properties of ^64^Cu-DOTA-anti-CTLA-4 mAb, and the data showed a significantly higher accumulation in the tumor [31]. Recent studies evaluated the biodistribution of ^64^Cu-labeled ipilimumab using PET imaging (Fig. 3), with a persistent high accumulation in CTLA-4-expressing lung cancer xenografts. Ipilimumab was found to bind to the CTLA-4-expressing tumor cells [32].

## Other immune checkpoints

### LAG-3

LAG-3 is a newly discovered immune checkpoint target expressed by activated T lymphocytes, which reduced T cell function [33]. LAG-3-positive TILs were significantly correlated with large mass volume of malignancies, high proliferation, and poor prognosis. The majority of PD-1/PD-L1-positive tumors concurrently show positive LAG-3 expression [34]. The application of multi-target IC blockade is a prospective treatment strategy for malignancies, and imaging of LAG-3 expression may provide evidence for the guidance of immunotherapy.

### Granzyme B

**Fig. 4:**
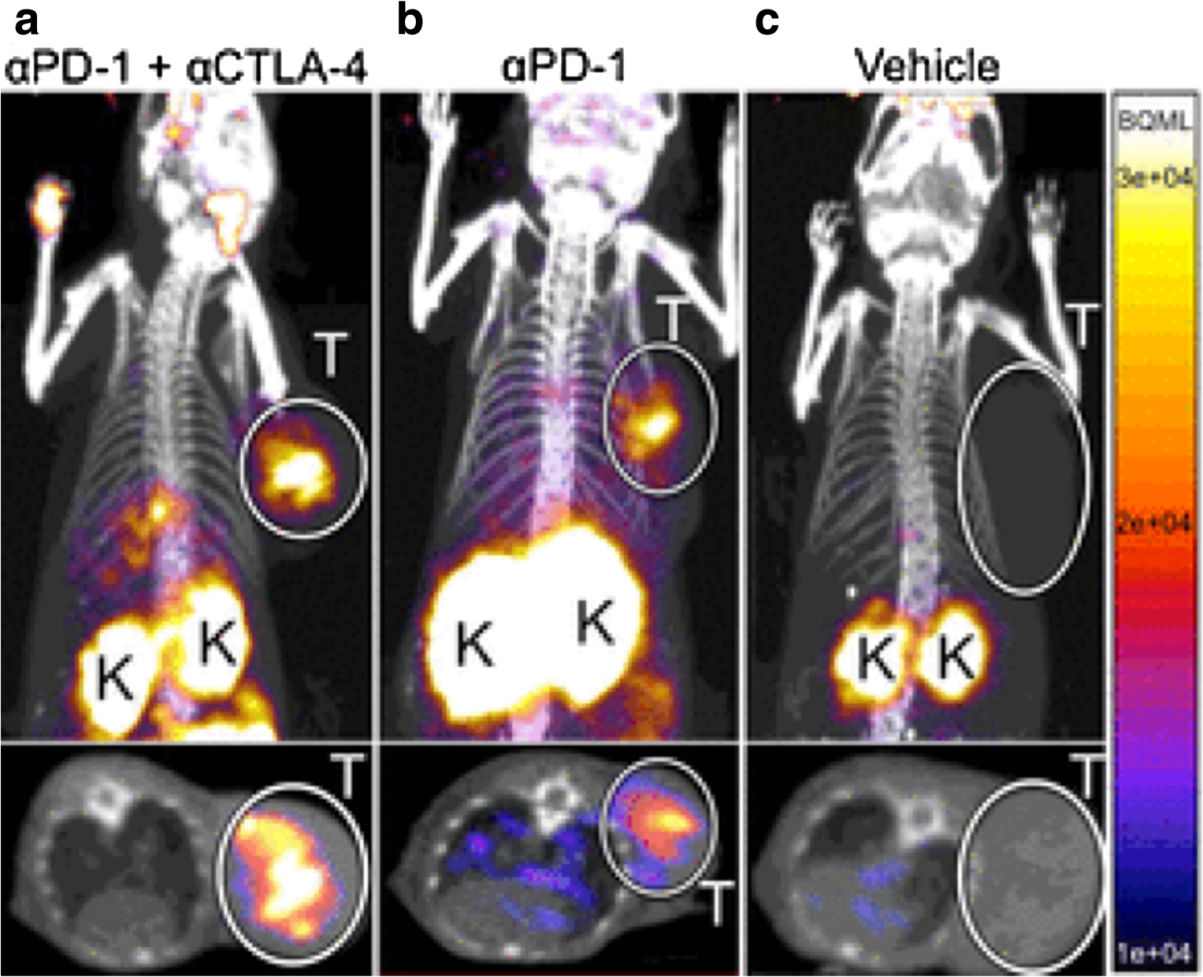
PET imaging of granzyme B following immune checkpoint blocking. The coronal and axial maximal intensity projection imaging of PET images of anti-PD-1 and anti-CTLA-4 combination-treated (**a**), anti-PD-1-treated (**b**), and vehicle-treated (**c**) colon tumor-bearing mice acquired 1 h postinjection of ^68^Ga-NOTA-GZP. The PET imaging showed high radionuclide signal intensity with ^68^Ga-NOTA-GZP in tumor with combination treatment of anti-CTLA-4 and anti-PD-1 antibodies. T tumors, K kidneys. (From Larimer BM, with permission of [35])

Checkpoint blocking treatment causes a higher expression of granzyme B, a reliable early-response biomarker for immunotherapy. The study utilized a novel probe ^68^Ga-NOTA-GZP to detect granzyme B using PET imaging [35]. Granzyme B is a serine protease released by active tumoral cytotoxic T cells. The immune synapses release granzymes, perforins, and granulysins to the synaptic cleft while binding to the tumor cells. Granzymes and perforins are death-inducing proteins causing the apoptosis of tumor cells [36]. Granzyme B-specific PET imaging was closely associated with granzyme B expression in CT26 colon tumors. The study reported higher radionuclide signal intensity with ^68^Ga-NOTA-GZP in tumor with combination treatment of anti-CTLA-4 and anti-PD-1 antibodies, compared to mono immune checkpoint therapy and untreated mice. It was proved that the probe can be utilized to differentiate nonresponders from responders who are suitable for immune checkpoint therapy in a sensitive manner [35] (Fig. 4). Moreover, the study also used an anti-human granzyme B antibody to analyze the human melanoma tumor of patients treated with anti-PD-1 immune checkpoint therapy by immunohistochemistry (IHC). It provides a promising clinical translation for targeted immune checkpoint imaging.

## Conclusion and future perspectives

Until now, only a fraction of receptors and ligands inhibiting immune responses have been identified and imaged. New generation of IC targets are potentially functional for malignant tumor therapy, such as OX40 (tumor necrosis factor receptor superfamily, also known as CD134), IDO, and TIM3 (T cell immunoglobulin and mucin domain-containing-3). Further imaging studies are needed to explore and monitor their dynamic expression in vivo and also immunotherapeutic effects more accurately. Imaging of immune checkpoint targets may provide further insight into immune therapeutic mechanisms, and is needed for the clinical translation. Moreover, the development of new and more functional imaging techniques is urgently needed to more precisely identify the expression of IC at the molecular and cellular levels. Imaging is helpful for the early diagnosis, cancer staging, and therapeutic effect evaluation. The advent of the new imaging modalities such as magnetic particle imaging (MPI), photoacoustic imaging (PAI), and the combination of several imaging methods is promising for immune-targeted imaging in patients with cancer. Last but not the least, most current studies of molecular imaging of IC targets are still at preclinical stage, and we can expect that clinical trials will develop and multiply in the near future. For this reason, and because we can anticipate that imaging will be a major tool for the evaluation of immunotherapy, imaging specialists need to become familiar with the mechanism of IC and the development of specific drugs, as well as with the technical implementation that will help them to play an important role in this rapidly evolving field.

## Abbreviations

CTLA-4: Cytotoxic T lymphocyte-associated protein 4
IC: Immune checkpoint
LAG-3: Lymphocyte activation gene 3
mAbs: Monoclonal antibodies
PD-1: Programmed cell death receptor 1
PD-L1: Programmed death ligand 1
TIL: Tumor-infiltrating lymphocytes
TREGS: Regulatory T cells
TNBC: Triple-negative breast cancer
